# Management of Subclinical Hyperthyroidism

**DOI:** 10.5812/ijem.3447

**Published:** 2012-04-20

**Authors:** Silvia Santos Palacios, Eider Pascual-Corrales, Juan Carlos Galofre

**Affiliations:** 1 Department of Endocrinology and Nutrition, University Clinic of Navarra, University of Navarra, Pamplona, Spain

**Keywords:** Hyperthyroidism, Disease Management, Therapeutics, Graves’ Disease

## Abstract

The ideal approach for adequate management of subclinical hyperthyroidism (low levels of thyroid-stimulating hormone [TSH] and normal thyroid hormone level) is a matter of intense debate among endocrinologists. The prevalence of low serum TSH levels ranges between 0.5% in children and 15% in the elderly population. Mild subclinical hyperthyroidism is more common than severe subclinical hyperthyroidism. Transient suppression of TSH secretion may occur because of several reasons; thus, corroboration of results from different assessments is essential in such cases. During differential diagnosis of hyperthyroidism, pituitary or hypothalamic disease, euthyroid sick syndrome, and drug-mediated suppression of TSH must be ruled out. A low plasma TSH value is also typically seen in the first trimester of gestation. Factitial or iatrogenic TSH inhibition caused by excessive intake of levothyroxine should be excluded by checking the patient’s medication history. If these nonthyroidal causes are ruled out during differential diagnosis, either transient or long-term endogenous thyroid hormone excess, usually caused by Graves’ disease or nodular goiter, should be considered as the cause of low circulating TSH levels.

We recommend the following 6-step process for the assessment and treatment of this common hormonal disorder: 1) confirmation, 2) evaluation of severity, 3) investigation of the cause, 4) assessment of potential complications, 5) evaluation of the necessity of treatment, and 6) if necessary, selection of the most appropriate treatment.

In conclusion, management of subclinical hyperthyroidism merits careful monitoring through regular assessment of thyroid function. Treatment is mandatory in older patients (> 65 years) or in presence of comorbidities (such as osteoporosis and atrial fibrillation).

## 1. Background

### 1.1. What is Subclinical Hyperthyroidism?

Subclinical hyperthyroidism is characterized by circulating thyrotropin (thyroid-stimulating hormone; TSH) levels below the reference range and normal serum thyroid hormone levels (1). The diagnosis is primarily biochemical and depends on the definition of “normal” TSH levels. In 2002, a panel of experts established the reference range for serum THS levels between 0.45 and 4.5 μU/mL (National Health and Nutrition Examination Survey [NHANES] III) (2) However, many specialists have proposed a reconsideration of this reference range in the light of data suggesting that, under certain circumstances, serum THS levels less than 4.5 μU/mL could mask thyroid dysfunction (3-9). Therefore, various aspects relevant to clinical practice should be considered in this regard. First, the traditional “one size fits all” policy cannot be applied to fix the reference range of circulating thyroid hormone levels. While interpreting serum TSH levels, physiological variations as well as presence of occult thyroid disease should be considered. Several anthropometric variables, including age, gender, race, and body mass index (BMI), have a noticeable influence over circulating TSH levels (8-10). In addition, other factors such as concurrent medication, coexisting pregnancy, or concomitant diseases should be considered in order to correctly interpret TSH, thyroxin (T4), and triiodothyronine (T3) status (8). As mentioned earlier, the definitions of both subclinical and overt hyperthyroidism (abnormal TSH with high thyroid hormone levels) are primarily biochemical, because the signs and symptoms of hyperthyroidism are nonspecific and may be present in patients with subclinical disease and absent in those with overt disease, especially in the elderly population. Additionally, clinicians should consider 3 important facts while interpreting the results of thyroid function tests (11): 1) TSH secretion follows a circadian rhythm with higher values early in the morning and lowest value in the afternoon; 2) THS secretion is pulse-regulated; and 3) THS half-life is about 15 min.

### 1.2. How to Screen for Thyroid Disease?

Although systematic screening for subclinical thyroid dysfunction is not recommended by the 2004 American experts’ task force, identification of cases in high-risk populations is highly advisable (12). However, serum TSH determination is universally regarded as the best test for the initial assessment of thyroid dysfunction (13). The plausible biochemical findings in patients with subclinical thyroid disease range between mild and severe dysfunction. A classification system for subclinical hyperthyroidism has been proposed recently, which differentiates between low serum TSH levels (0.1–0.4 μU/mL; Grade I or mild) and suppressed TSH concentration (< 0.1 μU/mL; Grade II or severe) (14). Grade I subclinical hyperthyroidism is 3 to 4 times more common than Grade II subclinical hyperthyroidism. The risk of progression from Grade I to overt disease is very low. Conversely, about 2% to 5% of the cases of Grade II hyperthyroidism progress to clinical disease per year (14). In some cases, subclinical hyperthyroidism may present with a normal serum level of free T4 (FT4) while the serum T3 level remains above the reference range. This unusual laboratory finding has been called “T3-toxicosis” and may represent the earliest stages of disease, which is normally caused by an autonomously functioning thyroid nodule (1). All these categories are relevant in clinical practice.

### 1.3. What is the Origin of Subclinical Hyperthyroidism?

Thyrotoxicosis can be exogenous or endogenous. Hyperthyroidism caused by underlying pituitary or hypothalamic disease should always be excluded during the differential diagnosis of subclinical hyperthyroidism (Sidebar).

**Sidebar:** Causes of Subclinical Hyperthyroidism (or Low Serum TSH Level)

Origin	Condition
**Endogenous**	
**Persistent**	Toxic Adenoma
	Toxic Multinodular Goiter
	Graves' disease
	Pituitary disease (Central hypothyroidism) a
**Transient**	Subacute thyroiditis
	Silent thyroiditis
	Postpartum thyroiditis
	Euthyroid Sick Syndrome
	Initial post-therapy period after treatment for overt hyperthyroidism
**Other**	Pregnancy (especially during the first trimester)
**Exogenous**	
**Iatrogenic**	Overtreatment with levothyroxine (most common cause)
	Factitial thyrotoxicosis (surreptitious levothyroxine intake)
	Drug-induced thyroiditis (amiodarone, α-IFN)
	Iodide excess (radiographic contrasts)
	TSH-lowering medications (steroids, dopamine)

^a^Usually associated with low Thyroxin and low Triiodothyronine

Exogenous subclinical thyrotoxicosis: This is the most common type of subclinical hyperthyroidism. Irrespective of the cause of hypothyroidism, thyroid blood tests in overtreated hypothyroid patients show results consistent with those of subclinical hyperthyroidism. Similar findings are observed in many patients with a history of differentiated thyroid cancer, in which the therapeutic target is to achieve and maintain TSH suppression by administering supraphysiological doses of levothyroxine (LT4). Thus, factitial or iatrogenic THS suppression due to excessive intake of LT4 should always be excluded by history-taking during the differential diagnosis of subclinical hyperthyroidism.

Endogenous hyperthyroidism: Subclinical overactive thyroid dysfunction similar to that in overt hyperthyroidism may be caused by Graves’ disease, toxic multinodular goiter, toxic adenoma, and different types of thyroiditis (15).

Miscellaneous conditions: TSH secretion may be transiently suppressed because of other causes such as euthyroid sick syndrome or concomitant use of TSH-suppressing drugs, e.g., steroids, dobutamine, amiodarone, and dopamine. These conditions are usually associated with low or low–normal serum T4 and T3 levels (16), and therefore, should be ruled out in the differential diagnosis of subclinical hyperthyroidism. Moreover, low TSH levels are also normally seen in the first trimester of pregnancy (17).

## 2. Patients and Methods

**Figure 1:**
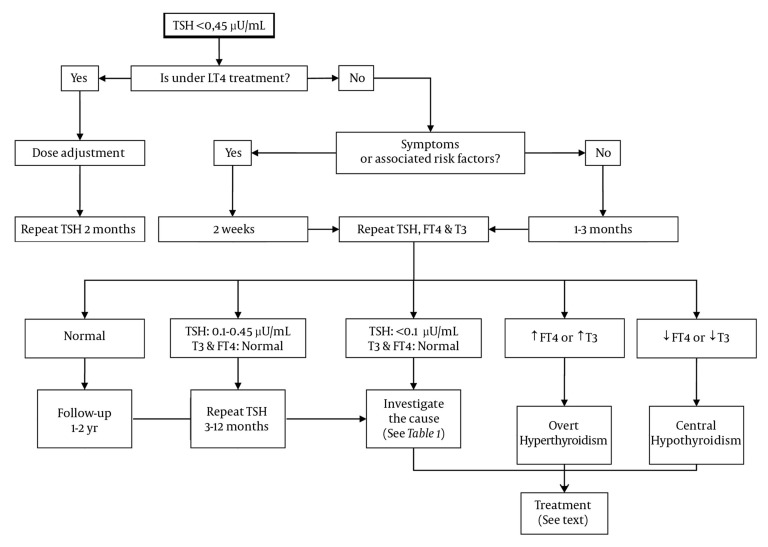
Flowchart for Management of Subclinical Hyperthyroidism

After the diagnosis of subclinical hyperthyroidism, management of the condition involves the following 6 steps: 1) confirmation, 2) assessment of severity, 3) determination of the cause, 4) assessment of potential complications, 5) evaluation of the necessity of treatment, and 6) selection of the most convenient therapy, in patients who require treatment. A flowchart summarizing this process is displayed in Figure 1. These steps should be followed for all patients, irrespective of their age, although elderly patients should receive thorough management.

### 2.1. Confirmation of the Diagnosis of Subclinical Hyperthyroidism

A case showing low serum TSH concentration with normal levels of peripheral thyroid hormones in absence of symptoms requires further confirmation with a complete thyroid profile analysis, including assessments of serum FT4 and T3 levels, after 1–3 months. If symptoms (such as cardiac arrhythmias) suggestive of hyperthyroidism are present, TSH measurement should be repeated after 2 weeks (18). Some reports indicate that approximately 50% of the cases of abnormal serum TSH levels resolve spontaneously, especially in Grade I (9-21). Spontaneous remission is more likely in subclinical Graves’ disease than in toxic multinodular goiter; accordingly, subclinical hyperthyroidism due to autonomous nodule(s) is more likely to progress to overt hyperthyroidism than that related to Graves’ disease (22). Patients showing consistently low TSH levels in repeated measurements for over a 3–6-month period should be diagnosed as having a thyroid disorder (1).

### 2.2. Severity Assessment

Patients showing persistently very low serum TSH values (< 0.1 μU/mL; Grade II) should be treated for the underlying cause of subclinical hyperthyroidism. Treatment is mandatory in patients aged over 65 years and in patients with associated comorbidities (such as heart disease or osteoporosis) or symptoms suggestive of hyperthyroidism (see section 5). A confirmed finding of mild to low serum TSH values (0.1–0.4 μU/mL; Grade I) indicates that treatment may be not necessary (23). However, careful monitoring of this group of untreated patients is recommended. Periodic assessment should include measurement of TSH, FT4, and T3 levels every 6 months. In contrast, symptomatic, elderly patients (> 65 years), and patients with underlying cardiovascular diseases should always receive appropriate treatment. Additionally, treatment is also recommended for patients who show a positive thyroid radionuclide scan with 1 or more focal areas of increased uptake (evidence of autonomy).

### 2.3. Determination of the Causes of Subclinical Hyperthyroidism

As mentioned earlier, TSH may be transiently suppressed because of various reasons (Sidebar). Investigation and confirmation of the cause are important steps during follow up. The most common cause of endogenous subclinical hyperthyroidism is release of excess thyroid hormone by the thyroid gland (1). In older persons, toxic multinodular goiter is probably the most common cause of subclinical hyperthyroidism (24). Nevertheless, patients should be inquired about any current medication that they may be using because some frequently used drugs (such as steroids or exogenous T4) inhibit pituitary TSH secretion, especially in older patients. Serum TSH concentrations in treated hyperthyroid patients or in those recovering from the thyrotoxic phase of thyroiditis may remain low even several months after normalization of circulating T4 and T3 levels. Transient low serum TSH levels are considered normal during the first trimester of pregnancy. True subclinical hyperthyroidism may occur in pregnant women, but it is not typically associated with adverse outcomes during pregnancy and does not require therapy (25). Individuals with central hypothyroidism generally have low serum TSH levels and normal (but usually low or low–normal) FT4 and T3 levels. Some studies have reported the existence of an altered set point of the hypothalamic–pituitary–thyroid axis in some healthy elderly people (26, 27). Elderly people may have low serum TSH levels and low–normal serum levels of FT4 and T3, without evidence of thyroid or pituitary disease (28, 29).

### 2.4. Assessment of Potential Complications

Clinical manifestations of both overt and mild (or subclinical) thyrotoxicosis are similar, but differ in magnitude. Potential complications of untreated subclinical hyperthyroidism are numerous and include weight loss, osteoporosis, atrial fibrillation, embolic events, and altered cognition. The most profound consequences of subclinical overactive thyroid dysfunction are observed on the cardiovascular system (30) and the skeleton (16). The following potential complications are of utmost importance in elderly patients.

Bone and mineral metabolism: Thyroid hormones directly stimulate bone resorption. Changes induced by the thyroid hormones are mainly observed in the cortical bone (wrist), to a significantly low extent in the trabecular bone (spine), and to an intermediate extent in the mixed cortical–trabecular bone (hip). Whether subclinical hyperthyroidism increases the fracture rate in the normal population is a matter of intense debate (31). However, postmenopausal women with subclinical hyperthyroidism may have high fracture rates even though they show only mildly suppressed serum TSH levels (31, 32). Therefore, to rule out osteoporosis, it is advisable to include a bone mineral densitometric study in the assessment of these patients.

Cardiovascular effects: Subclinical hyperthyroidism has several negative effects on cardiac function. These effects are similar to, but milder and less frequent than, those of overt hyperthyroidism. In a recent population-based study in Scotland, endogenous subclinical hyperthyroidism was associated with an increased risk of nonfatal cardiovascular disease (33). Additional related complications included tachycardia, atrial fibrillation, increased left ventricular mass index, increased cardiac contractility, impaired endothelial function, reduced exercise tolerance, reduced heart rate variability, and hypercoagulability (34, 35). The presence of cardiovascular complications is probably dependent on the degree of TSH suppression, the underlying cause of hyperthyroidism, and the individual’s sensitivity to excess thyroid hormone. Small-scale uncontrolled studies have shown an improvement in cardiac parameters of patients after restoration of the euthyroid state (36, 37) or after treatment with beta-adrenergic blocking drugs (38). Atrial fibrillation is a well-recognized complication of hyperthyroidism that commonly leads to embolic events. The frequency of atrial fibrillation is high in patients with subclinical hyperthyroidism. A prospective cohort study in approximately 2,000 adults (age > 60 years), with a follow-up period of 10 years, showed that low serum TSH level is a risk factor for atrial fibrillation (39). This study analyzed the relationship between serum TSH levels and the incidence of atrial fibrillation. Cumulative incidence rates of atrial fibrillation in subjects with serum TSH values lower than 0.1 μU/mL (Grade II subclinical hyperthyroidism), between 0.1 and 0.4 μU/mL (Grade I subclinical hyperthyroidism), and within the normal range were 28%, 16%, and 11%, respectively. Analogous results were published in another prospective cohort study that showed a relationship between subclinical hyperthyroidism and development of atrial fibrillation, although the data did not support the hypothesis that unrecognized subclinical hyperthyroidism is related to cardiac disorders (40). Cardiovascular mortality as a complication of subclinical hyperthyroidism is a matter that requires detailed investigation. All-cause and cardiovascular mortalities were assessed in a 10-year prospective study in a population with low serum TSH level (41). The investigators found higher mortality rates at 1, 2, and 5 years of follow up, but not after 10 years. A recent meta-analysis of 5 population-based studies on subclinical thyroid dysfunction showed that the risk of cardiovascular mortality was not significant in the population with low serum TSH level (42). In contrast, another meta-analysis with a similar design showed that subclinical thyroid disease is associated with an increased risk of all-cause mortality (43). Overall, the increased risk of mortality from subclinical hyperthyroidism appears to be low, but it could increase with age and degree of TSH suppression (43).

Dementia and depression: Although the data are conflicting, some authors have suggested that subclinical hyperthyroidism may be associated with dementia (33, 44). In contrast, a cross-sectional study in 295 English patients diagnosed with subclinical thyroid dysfunction did not show any association between subclinical hyperthyroidism and depression, anxiety, or cognitive function (45).

### 2.5. Need for Treatment

In endocrinology, the necessity of treatment for patients with subclincal hyperthyroidism is an open question (31, 46-50). The criteria for treatment of this disorder are controversial, and individualized judgment is mandatory in order to evaluate the grade and clinical consequences of the disorder in a given patient. Since prospective studies show that isolated low serum TSH levels spontaneously return to normal in nearly 50% of patients, caution and regular monitoring are the recommended initial approaches (31, 46). Additionally, only 5% of individuals with subclinical disease develop overt dysfunction yearly.

The 2004 American guidelines classified subclinical hyperthyroid patients according to the origin (endogenous or exogenous) and severity of the disorder. According to these guidelines, the treatment depends on the above mentioned criteria and should be decided after considering the risks and benefits in each case (31).

In any event, therapy strategies vary depending on 3 key factors (47, 49, 50): 1) cause, 2) severity, and 3) associated morbidity.

1. Cause: Etiological diagnosis must be performed before the initiation of treatment.

Exogenous subclinical hyperthyroidism in patients receiving LT4 treatment only requires dose adjustment (31). However, it is necessary to consider that certain patients with a medical history of thyroid carcinoma need chronic TSH suppressive therapy in order to maintain undetectable circulating TSH levels.

Subclinical hyperthyroidism in patients with Graves’ disease should be medically treated, although experimental data supporting this approach are limited (31). However, a number of patients with mild Graves’ disease develop spontaneous remission without therapy. Therefore, periodic monitoring of thyroid function every 3 months, without initiation of therapy, is a reasonable approach in young patients with mild subclinical hyperthyroidism caused by Graves’ disease (1). In contrast, subclinical hyperthyroidism caused by nodular hyperthyroidism usually requires ablative treatment because spontaneous normalization of thyroid function in this condition seldom occurs. Thus, surgery or administration of radioactive iodine is the treatment of choice in such cases.

The thyrotoxic phase of thyroiditis is transitory, with a middle-time (2 to 3 months) spontaneous remission, and usually needs no treatment or symptomatic treatment at the most (51).

2. Severity: In general, patients with Grade I subclinical hyperthyroidism may not be offered treatment. However, treatment of Grade I subclinical hyperthyroidism is warranted in elderly patients (> 65 years), in patients with cardiovascular disease, osteoporosis, and symptoms of hyperthyroidism, and postmenopausal women (especially those who have not been treated with estrogens or bisphosphonates) (1). Nevertheless, data regarding the potential benefits of treatment in these patients are inconclusive. Restoration of serum TSH levels contributes to bone mass preservation (52-54); however, normalization of bone turnover rates may require several months (47, 49, 50). A recent meta-analysis suggested a progressive increase in mortality rates of people aged over 60 years with subclinical thyroid dysfunction (43). However, the 2004 American experts’ task force recommends against routine treatment of patients with Grade I subclinical hyperthyroidism because the treatment neither benefits heart function nor improves arrhythmia in these patients (31). After careful consideration, the 2005 Joint Statement from the American Association of Clinical Endocrinologists concluded in favor of monitoring this group of patients (46). On the other hand, treatment should be strongly considered in high-risk individuals with persistent endogenous Grade II (TSH level < 0.1 μU/mL) subclinical hyperthyroidism (14).

Notwithstanding the recent 2011 Guidelines, there are still insufficient data to recommend for or against treatment of subclinical hyperthyroidism in young people with subclinical hyperthyroidism. A study on middle-aged patients showed an improvement in hyperthyroidism symptoms after treatment (36). However, in spite of the fact that the study did not include young people, the recent American Guidelines recommend treating all patients younger than 65 years with persistent and symptomatic Grade II subclinical hyperthyroidism (1).

3. Associated morbidity. As has been repeatedly indicated, the potential harmful effects associated with subclinical hyperthyroidism are the major reasons for starting specific treatment, as was recommended by the European Guidelines (48). These side effects are more evident in the elderly people and in patients with associated risk factors (48). Therefore, it is important to evaluate whether the following specific clinical situations are present at diagnosis: 1) hyperactive thyroid dysfunction of nodular origin; 2) goiter; 3) symptoms of thyrotoxicosis (generally nonspecific, such as fatigue, diarrhea, or palpitations); 4) cardiovascular disease (such as atrial fibrillation, angina, or heart failure); 5) bone or neuromuscular conditions; 6) gonadal dysfunction (oligomenorrhea, amenorrhea, or infertility); 7) old age (≥ 65 years); 8) circulating T3 levels in the upper limit of the normal range; and 9) very low serum TSH levels (< 0.01 μU/mL or severe Grade II).

### 2.6. Election of Treatment

The armamentarium for treatment of thyroid dysfunction is similar for overt and subclinical disease: medical therapy (antithyroid drugs and beta-adrenergic blockers), radioiodine administration, or surgery. The rationale and aim of subclinical hyperthyroidism therapy is to restore the euthyroid state and avoid potential side effects (36).

Medical therapy: Antithyroid drug therapy (carbimazole or its metabolite methimazole) at low doses (5–10 mg/day) is the treatment of choice in patients with Graves’ disease. Treatment should be continued, under adequate monitoring, for at least 6 months. Long-term treatment (over 12 to 18 months) is a sensible initial alternative to radioiodine therapy, because the remission index is high in patients with mild disease, especially in young people (55). Carbimazole-related adverse effects are usually not severe and can normally be managed by changing the dose of the drug, although exceptional severe events (such as agranulocytosis) have been described. Propylthiouracil could be an alternative option to carbimazole, but recent studies have warned practitioners about the use of this drug (56). Propylthiouracil should never be used in children (57) or in adults, except during early pregnancy when propylthiouracil may help avoid potential fetal malformations. In addition to causing liver disease, propylthiouracil is also associated with a greater risk of developing vasculitis and glomerulonephritis (58). Ablative treatment (surgery or radioiodine administration): Ablative therapy is generally safe and has no associated complications; however, ablation in asymptomatic patients is not advised. The presence of Graves’ orbitopathy normally precludes the administration of radioiodine. Ablation is the only definitive therapy for nodular hyperthyroidism (48). Surgical treatment is the recommended approach in young individuals with a single hyperfunctioning nodule (toxic adenoma) and in patients with toxic multinodular goiter (especially those showing compressive symptoms) or possible malignancy (1). Administration of radioactive iodine is appropriate for most patients, especially those with toxic multinodular goiter or over 60 years of age (1) and is the best treatment option in patients with concomitant heart disease. Either surgery or radioactive iodine administration may be recommended for patients with Graves’ disease if medical therapy fails.

Symptomatic treatment: Treatment with beta-blockers is recommended especially in the presence of adrenergic symptoms. Beta-blockers improve hyperthyroid symptoms and control cardiovascular morbidity, especially that caused by atrial fibrillation (38).
